# Attributional style in Borderline personality disorder is associated with self-esteem and loneliness

**DOI:** 10.1186/s40479-024-00263-2

**Published:** 2024-08-23

**Authors:** Anna Schulze, Berit Rommelfanger, Elisabeth Schendel, Hannah Schott, Aimée Lerchl, Ruben Vonderlin, Stefanie Lis

**Affiliations:** 1 Department of Clinical Psychology, Medical Faculty Mannheim, Central Institute of Mental Health, University of Heidelberg, Heidelberg, Germany; 2 Department of Psychosomatic Medicine and Psychotherapy, Medical Faculty Mannheim, Central Institute of Mental Health, University of Heidelberg, Heidelberg, Germany

**Keywords:** Attributional style, Self-esteem, Psychosocial functioning, Loneliness, Borderline Personality Disorder

## Abstract

**Background:**

Attributions are the processes by which individuals explain the causes of positive and negative events. A maladaptive attributional style has been associated with reduced self-esteem, psychosocial functioning, and mental health. Although many psychosocial interventions target an individual’s attributional style in mental disorders, studies of its alterations in Borderline Personality Disorder (BPD) are sparse. This study aimed to investigate the attributional style in patients with BPD in comparison to healthy control individuals (HC) and its association with self-esteem and psychosocial functioning.

**Methods:**

The participants (32 patients with a diagnosis of BPD, 32 HC, groups were balanced for sex, age and education) assessed their attributional style in regard to locus of control, stability and globality for positive and negative scenarios. Attributional style was compared between groups and linked to self-reports of self-esteem, loneliness and psychosocial functioning in different social domains while controlling for BPD and depressive symptom severity.

**Results:**

Individuals diagnosed with BPD reported a maladaptive attributional style for both positive and negative events. This was found to be strongly related with lower self-esteem and higher levels of loneliness, but not with psychosocial dysfunctions assessed in different social domains. The severity of BPD and depressive symptoms did not fully explain the association of attributional style with self-esteem and loneliness. In contrast, correcting for acute psychopathology actually strengthened the relationship between self-esteem and maladaptive inferring causality for positive events.

**Conclusion:**

The differential association of attributional style for positive and negative events with self-esteem and psychosocial functioning highlights the importance of considering the different facets of inferring causality during psychosocial interventions. Our findings suggest that the significance of cognitive alterations may change with remission of acute BPD and depressive psychopathology, depending on the valence of an event.

**Supplementary Information:**

The online version contains supplementary material available at 10.1186/s40479-024-00263-2.

## Background

Attributions are the processes by which individuals explain the causes of positive and negative events including the outcomes of social interactions and the behaviours of interaction partners [1, 2]. Individuals differ in their attributional, or explanatory, styles with consequences for their emotions, cognitions and behaviours [3]. Maladaptive attributional styles are trans-diagnostically relevant for psychosocial health [4]. Their relationship with depression and feelings of social isolation has been well-established across cultures for many years (see for example [5]). Maladaptive attributional styles are closely related to a low self-esteem and poor psychosocial functioning [6–8]. In contrast, a positive attributional style for positive events can function as a protective factor by weakening the association between negative life events and depression [9]. While a small number of studies have investigated the attributional style in Borderline Personality Disorder (BPD), their findings are inconsistent. In addition, it is unclear whether changes of these processes are related to the self-esteem and level of psychosocial functioning of individuals with BPD and how the severity of psychopathological symptoms affects these associations.

### Attributional styles

Depending on the specific theoretical model, attributions can be characterised along different dimensions. The most established approach distinguishes between the locus, stability and globality of the inferred cause (see for example [10, 11]). Locus refers to whether an individual locates the cause internally (i.e. to themselves), or externally (i.e., to others or circumstances). Stability and globality refer to whether an individual generalises the causes across time and situations, respectively. An individual’s attributional style has been linked with their self-esteem [6–8]. Healthy individuals mostly show a bias during inferring causality that serves the motive of self-enhancement [12]. That is, they attribute the causes of positive events as more global, stable, and internal than the causes of negative events. This so-called “self-serving bias” protects or even enhances an individual’s self-esteem. In contrast, individuals with a low self-esteem often reveal a maladaptive attributional style: they attribute negative events as caused by stable and global features of themselves, while explaining positive events as caused by others or circumstances and being restricted to a specific situation and point in time. Such an attributional style is assumed to pertain or even reduce an individual’s self-esteem and to result from the motive of self-verification, that is, the preference for explanations that confirm one’s own negative self-concept [13]. Such a maladaptive attributional style has been linked to negative self-conscious emotions (e.g., shame or guilt), motivational impairments and feelings of social isolation (see for example [14, 15]). A maladaptive attributional style constitutes a trans-diagnostic feature characterising individuals with mental disorders such as depressive disorders, schizophrenia and schizophrenia-spectrum disorders, attention-deficit-hyperactivity syndrome, autism or addictive behaviour (see for example [16–20]). It has been associated with a lower social competence and lower psychosocial functioning (see for example [21, 22]).

### BPD and attributional styles

BPD is a mental disorder affecting 0.7–2.7% of the general population and around 22% of in-patients in psychiatric hospitals [23–25]. Beyond affective instability and identity disturbances, the psychopathology of BPD is characterised by impairments in interpersonal relationships [26]. In the domain of self-functioning, several studies revealed alterations in cognition, emotions and motives of self-referential processing that are linked to a markedly low self-esteem in individuals with the diagnosis of BPD (for review see [27]). The negative view of one’s self is associated with impairments in trusting others and impairments in inferring mental and affective states of social interaction partners (for reviews see [28–31]).

Many cross-sectional and prospective studies revealed that the level of psychosocial functioning is consistently low in individuals with BPD (see for example [32]), that once achieved higher levels of functioning are not stable over time (see for example [33]) and that psychosocial functioning is not satisfactorily improved by disorder-specific psychosocial interventions (see for example [34]). Moreover, individuals with BPD report a reduced sense of belonging describing themselves as social outsiders, socially isolated and experiencing high levels of loneliness [35–38]. Loneliness is a negative affective state that arises from a mismatch between desired and perceived social connectedness [14]. Liebke et al. [36] found that it is related to psychosocial functioning in BPD. Loneliness can serve as a proxy for the subjective component of psychosocial functioning, which is a person’s reduced satisfaction with their social relationships.

Several studies described alterations in how patients with BPD infer causality using self-report scenario-based questionnaires to measure an individual’s attributional style. Moritz et al. [39], Schilling et al. [40], and Winter et al. [41] consistently identified a maladaptive attributional style in patients with BPD for negative events: patients with BPD attributed the cause of negative events to a higher percentage towards themselves compared with healthy control individuals. Similarly, Gutz et al. [42] found that BPD patients attributed being rejected by their co-players during an experimentally controlled virtual ball tossing game more strongly to themselves than healthy control individuals do.

Concerning the attributional style for positive events, the findings are inconsistent. Winter et al. [41] found a maladaptive attributional style with BPD patients attributing the cause of positive events to a lower extent to themselves. The study by Schilling et al. [40] showed a similar –albeit only trend level significant – alteration in BPD. In contrast, the data of Moritz et al. [39] suggested an opposite effect with BPD patients regarding themselves to a higher extent as the cause for positive events. However, this divergent finding might primarily be due to differences in the attributional style of the healthy control group: they revealed no self-serving bias in contrast to previous studies (see for example [40]) that, employed the same self-report questionnaire. Only the study by Winter et al. [41] investigated differences in the attributional dimensions of stability and globality between BPD patients and healthy control individuals. Their findings support a maladaptive attributional style in BPD for both negative and positive events. For negative events, attributions were not only changed towards a stronger internal causal inference, but also towards judging the causes as more global and more stable. In contrast, the causes for positive events were not only attributed less to one’s self, but were also assessed as more variable over time and specific to individual events.

Previous research suggests that self-esteem and psychosocial functioning are related closely to an individual’s attributional style. However, to our knowledge no study has so far investigated the association between these constructs for people with a diagnosis of BPD. Low self-esteem, impairments of psychosocial functioning and high loneliness have been well-established findings in many studies on BPD (see for example [32, 41, 43, 44]). Uncovering an association of these constructs with alterations in the attributional style would deepen our understanding of the cognitive mechanisms underlying these impairments in personal and psychosocial functioning in BPD. Thus, the aim of the current study was to investigate (1) differences in attributional style and the relevance of the three attributional dimensions internality, stability and globality between BPD and healthy control individuals, and (2) the association of the attributional style with self-esteem and (3) with loneliness and psychosocial functioning. We hypothesized that (1) BPD patients would show a more maladaptive attributional style for positive and negative events compared with healthy control individuals, and that a more maladaptive attributional style would be associated with (2) a lower self-esteem and (3) higher levels of loneliness as well as lower levels of psychosocial functioning.

## Methods

### Study design

This is a cross-sectional study investigating differences in the attributional style between individuals with a diagnosis of BPD and a healthy control group. Additionally, we analysed the association of attributional style with self-esteem, loneliness and psychosocial functioning while controlling for the severity of psychopathological symptoms of BPD and depression.

### Sample

Sixty-four participants filled out the questionnaires of the study. Thirty-two participants were diagnosed with a BPD. Thirty-two participants were healthy control individuals (HC).

General exclusion criteria were pregnancy, organic diseases of the brain and traumatic brain injuries, intellectual disability, epilepsy and current or past neurological diseases such as stroke, brain tumour, autism, or developmental disorders. BPD patients had to meet at least 5 of 9 DSM-5 criteria for BPD and have no comorbid occurrence of generalized anxiety disorder, schizophrenia, schizoaffective disorder, schizophrenia-like disorder, delusional disorder or bipolar I disorder. Clinical diagnoses were assessed by trained psychologists using the Structured Clinical Interview for DSM-IV Axis I Disorders (SCID-I, [45]) and the borderline section of the International Personality Disorder Examination (IPDE, [46]). HC had no acute or lifetime mental illness and no psychotropic medication.

We assessed borderline symptom severity with the Borderline Symptom List (BSL-23, [47], range 0–4 with higher scores indicating higher levels of BPD symptoms) and Borderline features with the Borderline Scale from the Personality Assessment Inventory (PAI-BOR, [48], German version VEI-BOR [49]; range 0–72, with higher scores indicating higher levels of BPD features). In addition, depressive symptom severity was assessed with the Beck Depression Inventory (BDI-II, [50], German version [51]; range: 0–63 with higher score indicating higher levels of depressive symptoms).

All participants gave their written informed consent before the start of the study. The study was approved by the Research Ethics Board II of the University of Heidelberg, Germany. Please note that these data were measured in the context of a larger project investigating the effects of a social partner providing external causes of social rejection in a virtual-reality environment, data from which are reported in a separate paper (Schulze et al., in preparation).

### Measurements

#### Attributional style questionnaire

We assessed the attributional style with the Attributional Style Questionnaire (ASF-E, German version by [52]). Participants were presented with eight positive and eight negative situations. Examples are ‘You meet a friend who compliments you on your appearance’ (positive scenario) and ‘A friend comes to you with a problem; you are unable to help him/her’ (negative scenario). For each of these situations, they had to assess on a 7-point Likert scale in two items each (1) the extent to which this cause lies within themselves or in other individuals or circumstances (locus/internality), how (2) stable or variable (stability) and how (3) global or specific (globality) the cause is. To facilitate interpretation, rating scores were transformed by subtracting 4 from each rating score resulting in scores varying between – 3 (external, variable and specific attributions) and 3 (internal, stable and global attributions). An individual’s attributional style is the average score across the three dimensions calculated separately for positive and negative scenarios. Higher positive scores indicate a more internal-stable-global attributional style, which is maladaptive for negative events and adaptive for positive events. Lower negative scores correspond to an external-variable-specific attributional style, which is maladaptive for positive events but adaptive for negative events. Additionally, we calculated average ratings scores separately for each of the three dimensions.

#### Self-esteem

We measured self-esteem with the 10-item Rosenberg Self-Esteem Scale (RSES, [53]). Scores vary between 0 and 30, with higher scores indicating higher self-esteem. In this study, the RSES demonstrated excellent internal consistency (Cronbach’s α = 0.95).

#### Loneliness

We assessed the frequency, intensity and duration of loneliness with the 4-item version of the ULCA Loneliness Scale (ULS, [54]) as suggested by Qualter et al. [55] to capture different facets of loneliness. Each item was rated on a 5-point Likert scale (range 1–5). Rating scores were averaged for the four items resulting in scores for frequency, intensity, and duration ranging between 1 and 5. Higher scores indicate higher levels of loneliness. In this study, the subscales demonstrated good internal consistency for the frequency and questionable internal consistency for intensity and duration of loneliness (Cronbach’s α frequency α = 0.86, intensity α = 0.64, duration α = 0.61).

#### Psychosocial functioning

To capture psychosocial functioning in a broader context of different social domains, we used the Inventory of Psychosocial Functioning (IPF, [56]). The IPF is an 80-item, self-report measure of psychosocial functional impairment specifically developed for impairments in posttraumatic stress disorder. It focusses on measuring mental health-related impairment above physical health-related impairment. It assesses psychosocial functioning in multiple items rated on a 7-point Likert scale (0: never to 6: always) in seven subscales corresponding to seven social domains, that is, romantic relationships, family, work, friendship and socialising, parenting, education, and self-care. The IPF yields as total score the mean of all completed subscales. Higher scores indicate a greater functional impairment.

### Statistical analyses

Statistical analysis of the attributional style measured by the ASF-E was done by 2 × 2-ANOVA with the independent factor group (BPD vs. HC) and the repeated measurement factors ‘valence’ (positive vs. negative events). To analyse differences depending on the three dimensions of the attributional style, we extended this design to a 2 × 2 × 3-ANOVA by the additional repeated measurement factor ‘attributional dimension’ (‘internality’ vs. ‘stability’ vs. ‘globality’). Degrees of freedom in the ANOVAs were corrected according to Greenhouse-Geisser. Post-hoc comparisons were done with t-tests and Cohen’s d was reported as effect size (*p*-values of t-tests were Bonferroni-corrected for multiple comparisons). We investigated the association between the attributional style for positive and negative scenarios with self-esteem, loneliness (ULS for frequency, intensity, and duration) and psychosocial functioning in social domains (IPF) with Pearson’s correlation coefficients (one-tailed). Additionally, we explored by partial correlation coefficients the influence of BPD and depressive symptom severity as well as self-esteem on these potential associations in BPD. We chose α = 0.05 as the level of significance. All analyses were performed with IBM SPSS Statistics 29 (IBM, USA).

## Results

### Participants

The group of individuals with a diagnosis of BPD and the healthy control group were balanced for age, sex and education (sex: HC: 5 men, 27 women, BPD: 5 men, 27 women, χ^2^ = 0, *p* = 1; age: HC *M* = 28.2, *SD* = 8.1, BPD *M* = 29.4, *SD* = 12.7, *t* = − 0.458, *p* = .649; similar level of school education: Fisher’s exact test, two-sided, *p* = .526).

Compared with HC, BPD patients reported a higher severity of BPD symptoms (BSL-23: HC *M* = 0.17, *SD* = 0.16, BPD *M* = 2.06, *SD* = 0.95, *t* =-11.15, *p* < .001, *d* = -2.78), BPD features (VEI-Bor: HC *M* = 19.60, *SD* = 6.72, BPD *M* = 52.44, *SD* = 7.89, *t* = -17.94, *p* < .001, *d* = -4.48) and depressive symptoms (BDI: HC *M* = 4.69, *SD* = 4.12, BPD *M* = 33.59, *SD* = 11.26, *t* = -13.64, *p* < .001, *d* = -3.41). Eight (25%) of the BPD patients were free of psychotropic medication. Four (12.5%) of the BPD patients were diagnosed with a harmful use or dependency syndrome of alcohol or cannabis, 25 (78.1%) with a depressive episode or recurrent depressive disorder, four (12.5%) with a phobic anxiety disorder, one (3.1%) generalized anxiety disorder, one (3.1%) with an obsessive-compulsive disorder. Furthermore, thirteen (40.6%) were diagnosed with a post-traumatic stress disorder, three (9.4%) with an undifferentiated somatoform disorder, eight (25%) with an eating disorder, nine (28.2%) with an attention deficit disorder (seven with and two without hyperactivity), one (3.1%) with a tic disorder and three (9.4%) with a disorder of adult personality and behaviour.

### Attributional style

**Fig. 1:**
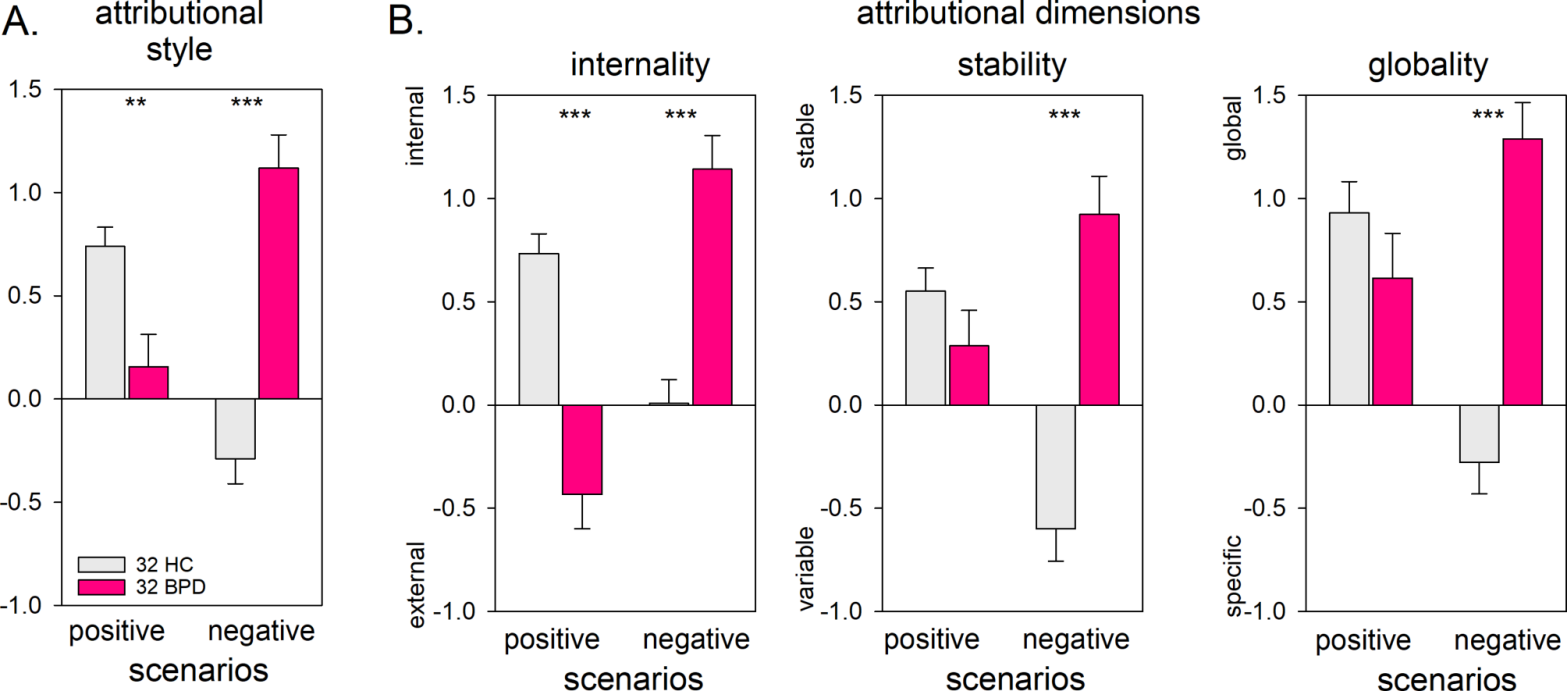
Attributional style and ratings of internality, stability and globality in positive and negative scenarios. Note: HC = healthy control group. Positive values (above the horizontal line) indicate a more internal-stable-global attributional style, negative values (below the horizontal line) indicate a more external-variable-specific attributional style

Attributional style differed between the BPD and HC group depending on the valence of the events (group * valence *F*(1,62) = 47.81, *p* < .001, *η*^*2*^_*p*_ = 0.435). In BPD, the attributional style was for positive events less internal-stable-global and for negative events more internal-stable-global compared with the HC group (Fig. 1A).

However, the strength of differences between groups differed depending on the attributional dimension and the valence of the event (group * valence * dimension (*F*(2,124) = 3.28, *p* = .042, *η*^*2*^_*p*_ = 0.05). For negative events, BPD patients assessed the causes as more internal, stable, and global compared with healthy control participants. In contrast, for positive events differences between groups were confined to the internality dimension with a more external attribution in the BPD group, but a more internal attribution in the HC group (Fig. 1B). The other dimensions did not reveal significant differences.

For tables of the rm-ANOVA results and pairwise comparisons between groups see supplementary material Table S1 and Table S2.

### Attributtional style and self-esteem

#### Self-esteem

BPD patients reported a lower self-esteem compared with healthy individuals (HC: *M* = 25.09, *SD* = 3.68, BPD: *M* = 10.47, *SD* = 4.77, *t* = 13.72, *p* < .001, Cohen’s *d* = 3.43).

#### Association between attributtional style and self-esteem

**Fig. 2:**
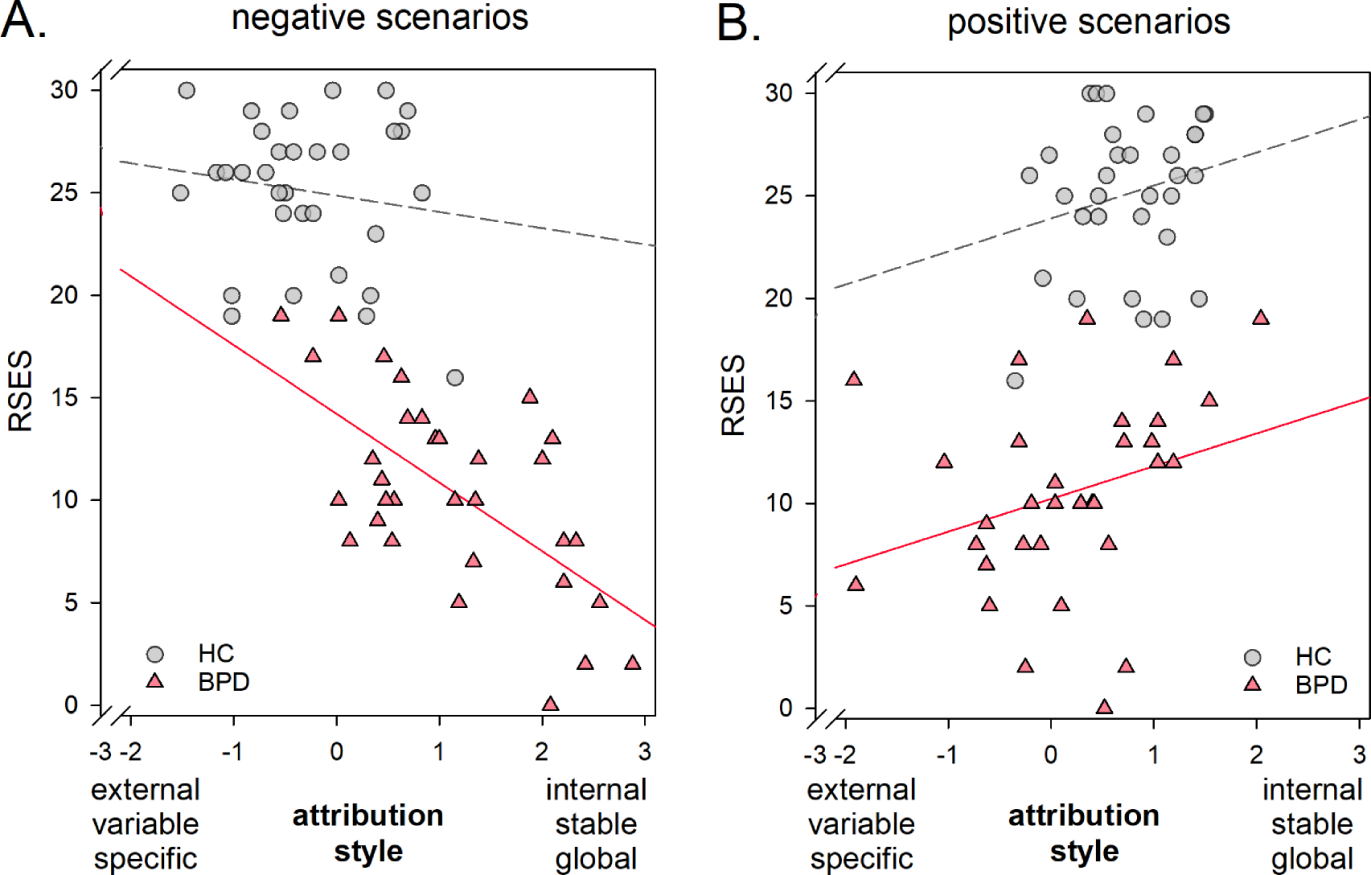
Association between attributional style in negative and positive scenarios with self-esteem. Note: positive values indicate a more internal-stable-global attributional style, negative values indicate a more external-variable-specific attributional style; circle correspond to healthy control individuals, triangles correspond to BPD patients

In BPD patients, a lower self-esteem was associated with a more internal, stable and global attributtional style for negative and a more external, variable and specific attributional style for positive scenarios (negative scenario: *r* = − .639, *p* < .001 indicating a large effect size; positive scenario: *r* = .300, *p* = .048, indicating a moderate effect size). The association of self-esteem and attributional style was significantly stronger for negative compared to positive scenarios (*z* = 1.74, *p* < .041, for comparison of absolute values of r). See Fig. 2A and B. This association was even observed after taking the severity of BPD psychopathology and depressive symptoms into account (partial correlation including BSL-23 and BDI scores as covariate for negative scenarios: *r* = − .506, *p* = .002; for positive scenarios: *r* = .575, *p* < .001). In contrast, statistical analyses revealed no significant correlation between self-esteem and attributtional style in the HC group (negative scenario: *r* = − .147, *p* = .210; positive scenario: *r* = .230, *p* = .103).

### Attributtional style, loneliness and psychosocial functioning

#### Loneliness and psychosocial functioning

BPD patients reported feeling lonely more often, more intense and for a longer duration compared with healthy individuals (ULS-frequency: HC: *M* = 1.84, *SD* = 0.66, BPD: *M* = 3.48, *SD* = 0.82, *t* = -8.82, *p* < .001, Cohen’s *d* = -2.21; ULS-intensity: HC: *M* = 2.27, *SD* = 0.65, BPD: *M* = 3.20, *SD* = 0.79, *t* = -5.18, *p* < .001, Cohen’s *d* = -1.29; ULS-duration: HC: *M* = 2.80, *SD* = 0.72, BPD: *M* = 3.67, *SD* = 0.53, *t* = -5.52, *p* < .001, Cohen’s *d* = -1.38).

The IPF score was higher in BPD patients compared with healthy control individuals indicating stronger impairments of psychosocial functioning (HC: *M* = 23.64, *SD* = 10.12, BPD: *M* = 49.67, *SD* = 10.87, *t* = -9.92, *p* < .001, Cohen’s *d* = -2.48).

#### Association between attributtional style and psychosocial functioning

**Fig. 3:**
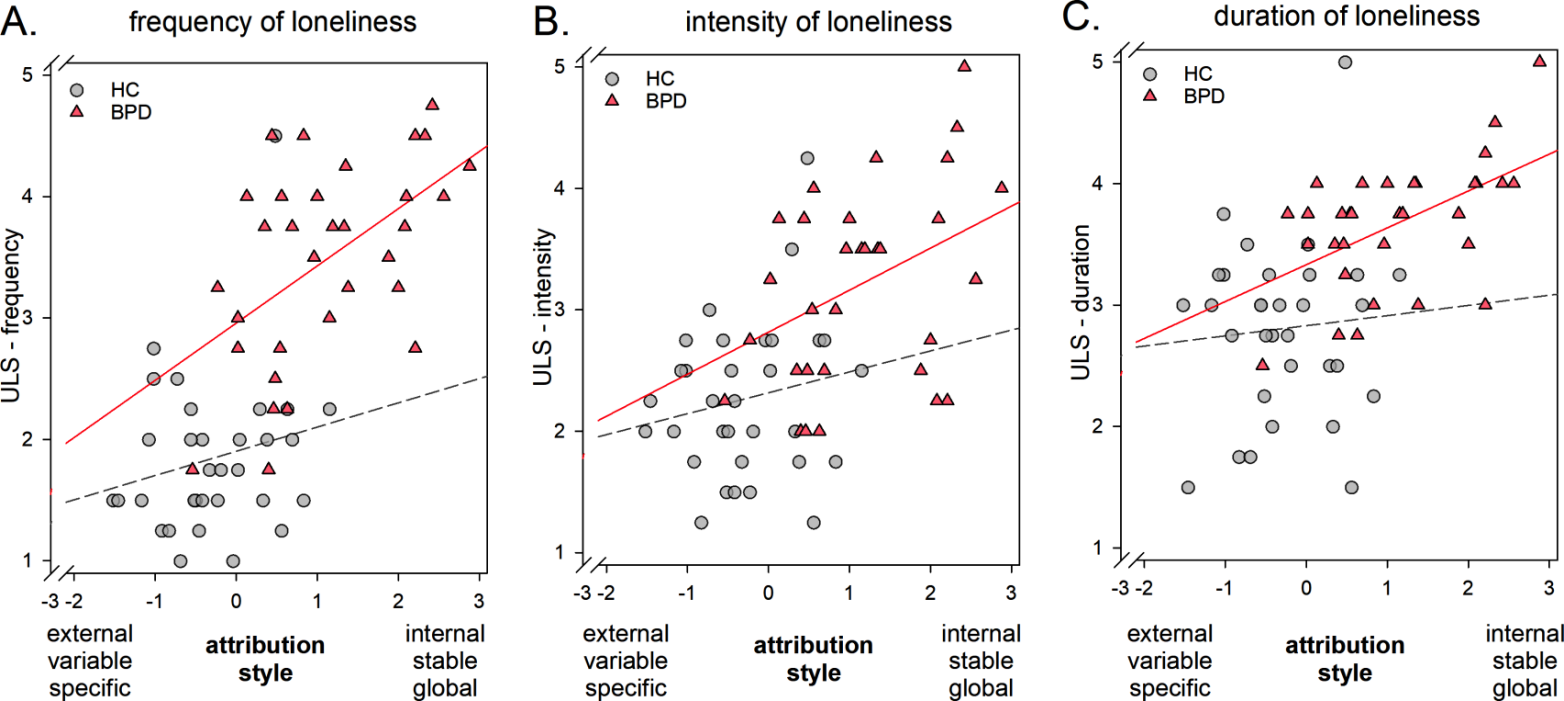
Association between attributional style in negative scenarios with frequency, intensity and duration of loneliness. Note: positive values indicate a more internal-stable-global attributional style, negative values indicate a more external-variable-specific attributional style; circle correspond to healthy control individuals, triangles correspond to BPD patients

In BPD patients, feeling more frequently, more intensely and for a longer duration lonely was associated with a more internal, stable and global attributtional style for negative, but not for positive scenarios (negative scenario: frequency *r* = .524, *p* = .001; intensity *r* = .397, *p* = .012; duration *r* = .527, *p* < .001; for positive scenario all *p*’s > 0.345). See Fig. 3. For the frequency of feeling lonely, this association was even observed after taking the severity of BPD symptoms, depressive symptoms and the level of self-esteem into account (partial correlation including BSL, BDI and RSES scores as covariate: *r* = .381, *p* = .021). In contrast, the associations with the intensity and duration of loneliness were only observed as a trend after controlling for psychopathology and self-esteem (partial correlation including BSL, BDI and RSES scores as covariate: intensity *r* = .264, *p* = .083, duration *r* = .286, *p* = .066).

In contrast in the HC group, a longer persistance of feeling lonely was associated with a more maladaptive attributional style for positive scenarios (*r* = − .403, *p* = .011) without an association with the frequency or intensity of loneliness or an association with the attributional style for negative scenarios (all *p*’s > 0.332). See supplementary material, Figure S1A.

For the BPD group, there was no correlation between the IPF score and the attributional style (for both positive and negative scenario *p*’s > 0.195). In contrast in HC, higher IPF scores indicating stronger psychosocial impairments were associated with a maladaptive attributional style for negative, but not positive scenarios (negative scenario: *r* = − .445, *p* = .005; positive scenario: *r* = − .190, *p* = .148). See supplementary material, Figure S1B.

## Discussion

The aim of the current study was to analyse differences in the attributional style in individuals with BPD compared with healthy control individuals and its associations with self-esteem, loneliness, and psychosocial functioning. Our results indicate a maladaptive attributional style in individuals with BPD, characterized by more external causal attributions for positive events and more internal, stable and global causal attributions for negative events. They extend previous studies by demonstrating associations of attributional style with self-esteem, as well as with loneliness as a proxy for a subjective facet of psychosocial functioning.

### Attributional style

In line with our hypothesis, individuals with BPD showed a maladaptive attributional style compared with healthy control individuals for both negative and positive events. Effect sizes suggested a stronger effect in negative than in positive scenarios. Groups differed in their attributions on the three dimensions depending on the valence of the event.

For negative events, individuals with BPD reported a maladaptive attributional style characterized by internal, stable, and global attributions. Consistent with previous studies, they attributed the underlying causes to themselves. In contrast, the healthy control individuals rated themselves and other people or circumstances as the cause of events to a similar extent [39–41]. Moreover, individuals with BPD generalised the causation more strongly over time and across different situations than healthy control individuals. These differences between groups confirm previous findings by Winter et al. [41] who conducted the only study that analysed the attributional dimensions of stability and globality.

In contrast, for positive events, individuals with BPD differed from healthy control individuals only on the locus dimension: BPD patients assessed the positive events as caused by other people or circumstances. In contrast, individuals in the HC group experienced themselves as responsible for the positive event. Effect sizes point to a strong effect, our finding is only partially in line with previous studies. Winter et al. [41] similarly observed a less internal attribution of positive events in BPD compared to HC. In line with this, Schilling et al. [40] found a similar difference between groups although only at trend level significance. The findings of the current study together with those of Schilling et al. [40] and Winter et al. [41] contradict the interpretation of Moritz et al. [39]. Moritz et al. assumed that an ego-centric perspective characterises the way individuals with BPD infer social causality. In contrast to the findings of Winter et al. [41], our data revealed no significant differences between groups in the attributional dimensions stability and globality: both groups tend to generalise the causes for positive events to a comparable extent as stable over time and across different situations. While the data of Winter et al. [41] suggest smaller effect sizes for stability and globality compared to internality, our data also revealed small effect sizes for both dimensions. However, these did not reach statistical significance in our sample.

In summary, our findings support a maladaptive strategy of inferring causality of both positive and negative events in BPD compared to healthy individuals. While the groups differed with regard to the locus of the cause for both positive and negative events a generalization across time and situations in BPD was specifically found for negative events. For negative and positive events, it is worth emphasizing that the rating scores for both groups vary in a rather restricted range. This might suggest that – at least at group level – the dichotomy in judging events internally or externally, stable, or variable and specific or global should be interpreted more as a tendency of judgments rather than dichotomous and extreme evaluations on the different dimensions. Nevertheless, alterations in the locus of control may interfere with the experience of personal agency during the course of treatment. One might speculate that particularly a reduced confidence in being able to positively influence one’s own life by decisions and choosing actions might prevent the attribution of therapeutic success to one’s own capabilities. This in turn may reduce motivation to invest efforts in changes. Previous research has shown a beneficial effect of an internal locus of control in the treatment of, for example, alcohol dependence, back pain and stuttering [57–59]. In line with this, Hashworth et al. [60] emphasized the role of personal agency, which relates to an internal locus of control, for post-treatment and 12 month follow up outcomes of dialectic behavioral therapy for BPD.

### Association of attributional style and self-esteem

A maladaptive attributional style has consistently been linked with lower self-esteem in previous studies [6–8]. Particularly an internal attribution of causes for positive events represents a cognitive self-serving bias: it has been linked to an individual’s need to stabilize and increase one’s self-esteem [12, 61]. In line with previous research (for review: [27], individuals with BPD in the current study reported a markedly lower self-esteem compared with HC individuals. In line with our hypothesis, an individual’s maladaptive attributional style was associated with a lower self-esteem in the BPD group. This was not the case in the HC group. Effect sizes point to a strong association with a shared variance of 41% for negative situations and a moderate association with a shared variance of 9% for positive situations in BPD. Importantly, these associations remained significant after taking the symptom severity of BPD psychopathology and depression into account. For negative situations, the association decreased slightly to 25% shared variance, suggesting that acute psychopathology partially explains the association between self-esteem and maladaptive attributional bias in negative situations. It seems worth noting that, in contrast, for positive events the association increased markedly to 33% shared variance when controlling for acute psychopathological symptoms. This indicates that acute psychopathological symptoms mask the link between self-esteem and a maladaptive attributional style when inferring causality for positive events. These findings might imply that the link between a maladaptive attributional style and low self-esteem for positive events persist even after remission from acute psychopathology hampering an enhancement of self-esteem by the lack of self-serving cognitive biases. Future research with symptom-remitted individuals with BPD is required to investigate whether a maladaptive attributional style, particularly for positive events, indeed persists after remission.

The results of our study indicate that maladaptive attributional style may be a relevant factor in clinical practice, even after symptom remission. Control expectations during psychosocial interventions, such as the belief in the ability to influence positive outcomes, have long been considered an important predictor of psychotherapy outcome (for a review see [62]). In line with this, changing locus of control attributions led to more adaptive attributions and job interview success in college students with low self-esteem [63]. Moreover, it improved emotion regulation in the context of negative social cues [64]. This emphasises maladaptive attributions as an important target for psychosocial intervention. Furthermore, maladaptive attributions may represent one of the cognitive mechanisms that contribute to impairments in the formation of positive relationships mediated by a low self-esteem. This may also affect the formation of a positive therapeutic alliance. For example, individuals with the diagnosis of BPD not only exhibited a particularly negative self-view, but also experience negative social feedback as more and positive social feedback as less applicable towards themselves [65]. Furthermore, a more negative self-view was not only associated with stronger BPD features but also a reduced preference for forming affiliations with others who provide positive feedback [66]. In light of our findings on the association between a maladaptive attributional style and low self-esteem in BPD, it can be posited that interventions targeting maladaptive attributional styles may prove beneficial in enhancing various aspects of the therapeutic process.

### Association of attributional style with loneliness and psychosocial functioning

Depending on the valence of the event, the pattern of the association between attributional style and psychosocial functioning varied. More specifically, an individual’s attributional style was differentially associated with loneliness as the subjective facet of impairments in psychosocial functioning and a broader assessment of psychosocial functioning in different social domains using the IPF in individuals with BPD and HC participants.

Individuals with BPD reported more intense feelings of loneliness compared with HCs. This is in line with previous studies [36, 37, 67]. Our data extend the literature by showing that individuals with BPD experience loneliness not only more intensely, but also more frequently and persisting for longer periods of time. In line with our hypothesis, feeling lonely more intensely, more frequently and for a longer duration was related to a maladaptive attributional style in BPD. However, these associations were restricted to infering causality for negative events. Only the frequency of loneliness was still related to the attributional style after controlling for symptom severity and self-esteem. This may suggest a differential relevance of the different facets of loneliness in BPD: while a more maladaptive attributional style may be a vulnerability factor for feeling socially disconnected more often after negative events, the ability to regulate the duration and intensity of this feeling may be particularly impaired in individuals with higher symptom severity. Although individuals with BPD reported a lower level of psychosocial functioning as assessed with the IPF compared with individuals of the HC group, attributional style did not co-vary with the level of psychosocial functioning measured by this broader assessment covering different social domains.

Our data suggest a differential pattern of associations of the attributional style with psychosocial functioning in healthy individuals depending on the valence of the situations as in individuals with BPD. In contrast to the BPD group, a longer persistence of loneliness was particularly associated with a maladaptive attributional style in positive situations. Contrarily, a maladaptive attributional style was associated with lower levels of psychosocial functioning in negative situations. However, this different pattern has to be interpreted with caution due to the overall low frequency of loneliness and impairments in psychosocial functioning in the HC group.

### Limitations, strengths, and future directions

Some limitations and strengths of the present study must be addressed.

Due to the rather small sample size, the results should be replicated in future studies with larger sample sizes. Another limitation is that women are overrepresented in our sample. This prevented the analysis of gender differences and might also require some caution in generalising our findings across sex and gender. Moreover, 75% of all individuals of the BPD group were treated with psychotropic medication. Thus, an influence of the different substances on cognitive biases cannot be excluded. Moreover, participants assessed their attributional style for different positive and negative scenarios using a self-report questionnaire. However, as already suggested by Schilling et al. [40], the experience of events, such as receiving a compliment, may differ between individuals with BPD and healthy control participants. Several studies revealed that the level of self-esteem might affect the evaluation of events as positive or negative: for example, receiving positive feedback from others, which would increase self-esteem in people with high self-esteem, may be experienced as false and cause anger or distrust and doubt about the honesty of others (see for example [68]). Furthermore, distinguishing between internal and external attributions can be challenging. For instance, Gutz et al. [42] found that individuals with BPD perceived exclusion from a virtual ball tossing game as caused by both themselves and hostile intentions of their co-players.

Although linking the inference of social causality with psychosocial functioning is, from our perspective, important and constitutes a strength of our study, we acknowledge that the IPF we used, has some disadvantages. Although it covers various social domains, it is important to note that some of these domains were not applicable to the individuals with BPD in the current study. For instance, some participants did not have a partner or children or were unemployed at the time of the study. The mean score across the applicable domains does not represent whether the absence of a subscale score may indicate additional psychosocial impairment. Future studies with larger sample sizes are needed to allow more differentiated analyses of psychosocial functioning in different social domains and their association with, for example, changes in cognitive processing like attributional style. Finally, our study is not suited to confirm a specificity of our findings for individuals with a diagnosis of BPD. Contrarily, cognitive biases in inferring causality are most probably of trans-diagnostic relevance (see for example [4]). Further studies with clinical control groups are required to investigate which alterations might be specific for individuals with a diagnosis of BPD or transdiagnostic relevant features of cognitive processing across various mental disorders.

Our study extends findings on impairments in the sense of belonging in people with BPD. This study is the first to show that the feeling of loneliness in BPD does not only differ in the intensity, but also in the frequency and duration of this aversive experience that threaten the fundamental need of humans to belong [69]. It has to be emphasised that the distinction between these three facets of loneliness is important, since we found differential associations with the attributional bias for positive and negative events differing for individuals with BPD and healthy control individuals. For example, the frequency of feeling lonely in BPD showed the strongest association with the attributional style towards negative events. However, in healthy control participants, the persistence of this feeling was linked with a maladaptive attributional style towards positive events. These distinct patterns across different facets of loneliness may contribute to the challenges in replicating findings, especially when using data from different countries. The ULS is among the most established instruments to measure loneliness. However, the German version assesses the intensity of loneliness, while the English version assesses the frequency. In consequence, differences between findings might be caused by measuring different facets of the construct, instead of reflecting cultural differences or issues with replicability. This might be particularly important when linking loneliness with other constructs such as cognitive biases. Our findings point to the relevance of measuring these different facets of loneliness in future studies to cover the construct more broadly and test whether the differential pattern we observed can be replicated in the future.

Our findings emphasise the relevance of cognitive biases for events with both a negative and a positive valence. This is in line with an increasing awareness that changes in social-cognitive processes in BPD are not restricted to a hypersensitivity for negative events, but also to alterations when evaluating positive events (see for example [70–71]). The differential relevance of the valence of events is also in line with studies showing that attributional processes differ depending on a positive and negative context in their underlying mechanism and consequences for well-being (see for example [72]). However, most importantly, the association of alterations in attributional processes in BPD were differentially associated with the severity of psychopathology for positive and negative events. Future studies are needed that address whether alterations in attributional processes in BPD are indeed differentially influenced by a remission of acute psychopathological symptoms. If this is the case, further research is needed to design and evaluate specific intervention components targeting attributional styles and their potential to enhance long-term self-esteem and psychosocial functioning as potential adjunctive interventions following the reduction of acute BPD symptomatology.

## Conclusion

Our data confirm a maladaptive attributional style in individuals with BPD. The current study goes beyond previous studies by revealing a close association of these cognitive processes with self-esteem and the feeling of loneliness as a proxy for a subjective aspect of psychosocial functioning. Differences in inferring causality towards positive and negative events suggest distinct valence-dependent mechanisms, which may also be differentially associated to remission of psychopathological symptom. Particularly maladaptive cognitive processes of evaluating the causes of positive events may persist in those with low self-esteem after remission from acute psychopathology. Further studies are needed that investigate the causal relationship between attributional style, self-esteem and loneliness in longitudinal studies and examine whether maladaptive attributional styles may constitute a vulnerability factor that persists after symptom remission and hinder a stable recovery during which individuals with BPD restore a sense of belonging within their social networks.

### Electronic supplementary material

Below is the link to the electronic supplementary material.

Supplementary Material 1

## Data Availability

According to European law (GDPR), data containing potentially identifying or sensitive patient information are restricted; our data involving clinical participants are not freely available in the manuscript, supplemental files, or in a public repository. Data access can be requested on reasonable demand via AS.
