# Supplementary material for: Attributional style in Borderline personality disorder is associated with self-esteem and loneliness
Source: Borderline Personal Disord Emot Dysregul. 2024 Aug 23;11:19. doi: 10.1186/s40479-024-00263-2 (PMC11342650; doi:10.1186/s40479-024-00263-2)

Supplementary Material

Attributional style in Borderline Personality Disorder is associated with self-esteem and loneliness

Schulze, A. [1*], Rommelfanger, B. [1], Schendel, E. [1], Schott, H. [1], Lerchl, A. [1], Vonderlin, R. [2], Lis, S [1, 2].

[1]Department of Clinical Psychology, Central Institute of Mental Health, Medical Faculty Mannheim, University of Heidelberg, Germany

[2]Department of Psychosomatic Medicine and Psychotherapy, Central Institute of Mental Health, Medical Faculty Mannheim, University of Heidelberg, Germany

Table S1

Results of the 2x2x3 rm-ANOVA

effect	$df_{1,2}$	*F*	*p*	η^2_p
group	1, 62	10.40	.002	.144
attribution dimension	2, 124	9.69	<.001	.135
attribution dimension * group	2, 124	9.86	<.001	.137
valence	1, 62	.05	.817	.001
valence * group	1, 62	47.81	<.001	.435
attribution dimension * valence	2, 124	27.62	<.001	.308
attribution dimension * valence * group	2, 124	3.28	.042	.050

Note: p's are reported for Greenhouse-Geisser corrected degrees of freedom

Table S2

Comparison between groups

attribution dimension	scenario	*t*(62)	*p*	95%CI		effect size Cohen's *d*
internality	positive	6.112	<.001	0.79	1.55	1.53
	negative	-5.681	<.001	-1.53	-0.74	-1.42
stability	positive	1.298	.199	-0.14	0.68	0.32
	negative	-6.293	<.001	-2.01	-1.04	-1.57
globality	positive	1.199	.235	-0.21	0.84	0.30
	negative	-6.689	<.001	-2.04	-1.10	-1.67

Note: Bonferroni corrected threshold is p<.008

Figure S1

Association between attributional style with duration of loneliness and interpersonal functioning

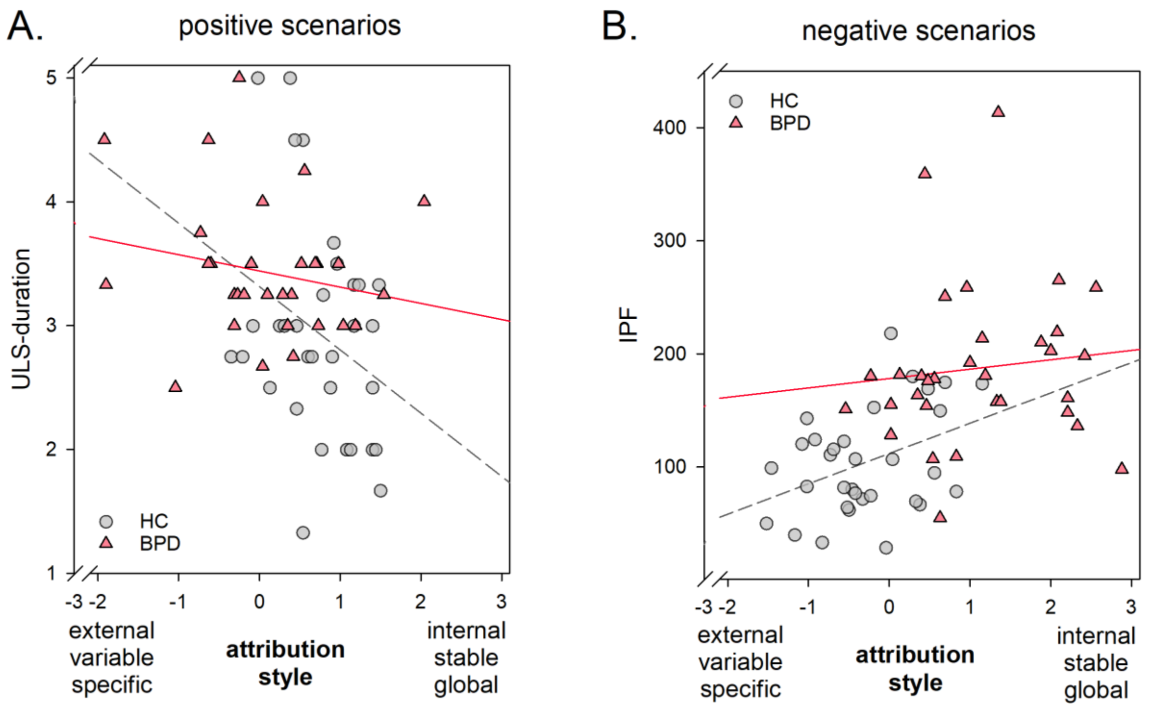

Supplement: Supplementary file 1 — Supplementary Material 1 [file 40479_2024_263_MOESM1_ESM.pdf]
